# Identifying modifiable factors associated with hospitalization outcomes in people with dementia residing in UK long‐term care homes

**DOI:** 10.1002/alz70858_099082

**Published:** 2025-12-24

**Authors:** Alba Fernandez‐Sanles, Gill Livingston, Connie Howard, Andrew Sommerlad

**Affiliations:** ^1^ Division of Psychiatry, University College London, London, Other, United Kingdom; ^2^ University College London, London, London, United Kingdom; ^3^ Division of Psychiatry, University College London, London, London, United Kingdom

## Abstract

**Background:**

Hospitalizations among people living in long‐term care (LTC) homes with dementia are common and often result in adverse outcomes, yet there is clear evidence for interventions which may be targeted at residents, staff and care home environments to reduce unnecessary hospitalizations. We aimed to explore factors associated with improved hospitalization outcomes for LTC residents with dementia which could be targeted in future interventions.

**Methods:**

This prospective cohort study uses data from the UK MARQUE study of UK LTC residents with dementia. Data was collected at baseline, and 4, 8, 12, and 16 months, on resident characteristics and symptoms, staff characteristics including training and burnout symptoms, and care home factors including size, and environment. The Client Services Receipt Inventory was used to collect number and duration of hospitalization for all participants at all time‐points. We will perform multivariable analyses to examine the association between a range of potential modifiable factors, including medication regimens, staffing characteristics, and care home features, and hospitalization outcomes.

**Results:**

We included 1,486 participants from 97 LTC homes. Seventy eight of the homes (80.4%) were privately managed and mean number of residents was 43.2 (SD = 21.4). Data were collected from 1,698 members of staff. Residents were predominantly female (1,026 (69%)), and older than 85 years old (804 (55.9%)). Residents had a range of dementia severity; 30% mild dementia, 33% moderate, and 37% severe). At baseline, 16.7% of residents were taking an antipsychotic, 39.6% were taking an antidepressant and 19.2% were taking a hypnotic or sedative.

**Conclusions:**

Identifying modifiable factors that influence hospitalization outcomes could guide interventions aimed at improving care and reducing hospital‐related complications in this vulnerable population. This research has the potential to shape future interventions and policies that improve the quality of life and health outcomes for people with dementia in care homes, with broader implications for healthcare systems managing aging populations.

